# Recovery of Diabetic Rats After Physical Exhaustion: Kinetic Alterations in Muscle Inflammation and Muscle-Signaling Proteins to Atrophy and Hypertrophy

**DOI:** 10.3389/fphys.2020.573416

**Published:** 2020-11-12

**Authors:** José Ricardo Bortolon, Gilson Masahiro Murata, Leandro Borges, Eleine Weimann, Maysa Braga Barros Silva, Alexandre Dermargos, Elaine Hatanaka

**Affiliations:** ^1^Instituto de Ciências da Atividade Física e Esporte (ICAFE), Universidade Cruzeiro do Sul, São Paulo, Brazil; ^2^Universidade Paulista (UNIP), São Paulo, Brazil

**Keywords:** immune system, skeletal muscle, interleukin, exhaustive exercise, cytokine, diabetes mellitus

## Abstract

The complexity of the adaptive response of diabetics to intense exercise is still poorly understood. To optimize exercise interventions in diabetics, the chronology of inflammatory mediators in muscle and the signaling involved in muscle hypertrophy/atrophy must be understood. Herein, we studied the kinetic inflammatory profile and cellular signaling pathways modulated by physical exhaustion after the induction of type 1 diabetes by streptozotocin in rats. Soleus muscle samples were obtained from diabetic and control groups at the following moments: baseline (no exercise); immediately after exhaustive exercise; and at 2 h, 24 h, 48 h, and 72 h after a treadmill exhaustive exercise. Kinetic production of cytokines and kinetic activation of proteins related to muscle synthesis (p70S6K and Akt) and degradation (GSK3, MuRF1, and MAFbx) were measured in the soleus muscle. We observed that the muscle TNF-α (0.9-fold; *p* = 0.0007), IL-1β (0.8-fold; *p* = 0.01), IL-6 (0.8-fold; *p* = 0.0013), L-selectin (1.0-fold; *p* = 0.0019), and CINC-2α/β (0.9-fold; *p* = 0.04) levels were higher in almost all stages of the study in the diabetic animals compared with the control group. Our data showed that exhaustive exercise decreased MAFbx expression in diabetic animals compared to the control group in a time-dependent manner. The decreased activation ratios of MAFbx were followed by a decrease in TNF-α, IL-1β, and IL-6 levels. p70S6k phosphorylation was also decreased in the diabetic group compared to the control group after physical exhaustion. Regarding the activation of proteins related to muscle synthesis and degradation, we found that the alterations induced by exhaustive exercise in the diabetic rats might involve pathways related to synthesis and muscle breakdown. Moreover, after an exhaustive exercise session, the recovery of the inflammatory response in the diabetic animals was slower than that in the control rats while the return of inflammatory cytokines to baseline levels was more effective in the diabetic animals.

## Introduction

The benefits of exercise as adjuvant therapy in the management of all diabetes types need to be constantly emphasized. Regular physical exercise reduces insulin resistance, reduces dyslipidaemia, regulates the endothelial role and hypertension, enriches motor control, and controls leukocyte function (Najafipour et al., 2017; Hasan et al., 2018). Nevertheless, information regarding the complexity of the adaptive response of diabetic subjects to intense physical activity is still scarce. Moreover, data on the time required by diabetic patients or animals with streptozotocin-induced diabetes to recover without risks from exercise-induced tissue damage are lacking (Bortolon et al., 2012). Such data are important because inflammation induced by intense physical activity may be detrimental to the health and muscle structure of patients with diabetes.

The difficulty in muscle repair in diabetic subjects is due to various factors, such as the existence of glycated proteins, changes in fatty acid levels, impairment of leukocyte functions, and subclinical chronic inflammation (Hatanaka et al., 2007; Richard et al., 2017). In addition to the constant subclinical inflammation, mitochondrial dysfunction, and skeletal muscle wasting are also common in individuals with diabetes. Atrophy is closely related to the inflammatory signaling pathways (Bonaldo and Sandri, 2013). Activation of the PI3K/Akt pathway blocks skeletal muscle atrophy and induces hypertrophy. The hypertrophic effects of Akt are modulated partly by stimulating protein synthesis pathways downstream of GSK3β and mTOR (Schiaffino and Mammucari, 2011), whereas the muscle-specific ubiquitin ligases MAFbx and MuRF1 are important for activating muscle atrophy. During muscle atrophy, MAFbx and MuRF1 bind to and mediate the ubiquitination of myofibrillar proteins for subsequent degradation. Few pharmacological targets have been provided in diabetic muscle atrophy studies, and information about post-exercise inflammatory management in diabetic muscle is relevant since both hypertrophy and atrophy depend on the inflammatory state generated by exercise (Schiaffino and Mammucari, 2011; Bonaldo and Sandri, 2013).

In the present research, we analyzed the initiation and resolution of muscle inflammation and tissue repair in the control and streptozotocin-induced diabetic animals submitted to a single session of exhaustive exercise. Measurement of pro-inflammatory cytokines in the soleus muscle [tumor necrosis factor (TNF)-α, interleukin (IL)-1β, IL-6, cytokine-induced neutrophil chemo-attractants (CINC), and L-selectin] was carried out before, immediately after, and at 2, 24, and 48 h after exercise. We also evaluated components of the signaling pathways Akt, MAFbx, P70S6K, GSK3, MuRF, and mTOR.

## Materials and Methods

### Experimental Animals

Male Wistar rats (180 ± 20 g) were maintained in an environment under controlled humidity and temperature conditions (inverted cycle of 12-h light/dark). Water and standard laboratory rats feed (21% proteins, 4% lipids, and 52% carbohydrates; Nuvilab CR1-Nuvital) were supplied *ad libitum*. The study was developed following the Guidelines for the Care and Use of Laboratory Animals. The animals were separated into a control group and a diabetic group and followed for five time points: (i) non-exercised (0), (ii) immediately after exercise (IA), (iii) 2 h after exercise (2 h), (iv) 24 h after exercise (24 h), and (v) 48 h after exercise (48 h).

### Induction of Diabetes

The induction of experimental type 1 diabetes (T1DM) was performed by intraperitoneal injection of 65 mg/kg body weight of streptozotocin dissolved in citrate buffer (pH 4.2). The diabetic status was determined 48 h after streptozotocin injection by blood glucose concentrations higher than 250 mg/dL, which were measured using a glucose meter (Roche, São Paulo, Brazil).

### Exercise Protocol

Seven days after the induction of diabetes, the animals were submitted to an exercise adaptation training program on a treadmill for 7 days. The treadmill adaptation was composed of daily exercise training for 15 min at a speed of 0.3 km/h (Belotto et al., 2010; Borges et al., 2015). One day after the conclusion of the adaptation period, the exercised animals (four rats per cage) were submitted to a unique session of exhaustive exercise. All the procedures were performed during the dark light phase.

The first speed of the treadmill was 0.3 km/h. The velocity was elevated by 0.3 km/h in 3-min intervals until the animals completed 20 min of exercise (Leandro et al., 2007; Bortolon et al., 2012). The criterion for exhaustion was a loss of motor control. Before, IA, and 2 h, 24 h, and 48 h after completion of the exhaustive exercise, the animals were euthanized and blood and muscle tissue were collected. Tissue (soleus muscle) was obtained and kept frozen (−80°C) before the assays.

### Tissue Collection and Sample Normalization

Tissue was collected from soleus muscle at 0 (IA), 2, 24, 48, and 72 h after the end of exhaustive exercise. For homogenization, phosphate-buffered saline was supplemented with protease inhibitors (0.5 M PMSF and 25 IU ml^–1^ aprotinin). Using a Polytron PT 3100 (Kinematica, Lucerne, Switzerland), the tissues (100 mg) were sonicated for 1 min and centrifuged (10 min, 1,000 × *g* at 4°C). Prior to the experiments in this study, the concentration of the total protein of each sample was measured by the Bradford method (Bradford, 1976).

### Determination of Cytokines

The cytokine levels of CINC-2α/β, IL-1β, TNF-α, IL-6, and L-selectin) were evaluated by ELISA (Duo Set kit, R&D System) as described by previous studies (Borges et al., 2014, 2019).

### Western Blot Analysis

Equal values of protein (150 μg/line) were resolved in SDS-PAGE and relocated to nitrocellulose membranes. Subsequently, the membrane was blocked for 2 h in a saline solution [10 mM Tris (pH 7.5), 150 mM NaCl, 0.05% Tween 20] at room temperature with 5% non-fat milk. Membrane washing (three washes, 10 min each) occurred in saline solution. Then, the membrane was incubated membrane with diluted primary antibody (Akt, mTOR, p70S6k, GSK3, and MAFbx) from Santa Cruz Biotechnology (Santa Cruz, CA, United States) in either 5% w/v BSA, 1X TBS, 0.1% Tween^®^ 20 at 4°C with gentle shaking, overnight. After a new membrane washing process, the membrane was incubated with an anti-IgG antibody (1:10,000 dilution) attached to horseradish peroxidase (saline solution with 1% non-fat milk) for 1 h. Posteriorly, the incubation of the membrane was performed using a substrate for peroxidase and a chemiluminescence enhancer (ECL Western Blotting System Kit, GE Health Care, Little Chalfont, Buckinghamshire, United Kingdom) for 1 min and submitted to X-ray film. After the development of the films, the intensity of the band was measured by optical densitometry (Hatanaka et al., 2013).

### Statistical Analysis

Except for the Western blot assays, the data are expressed as the means ± standard error (SE). The statistical analysis was performed via a repeated-measures two way (ANOVA) with Student-Newman-Keuls *post hoc* multiple comparison test (InStat; GraphPad Software, San Diego, CA, United States). The area under the curve was determined by the *t*-test. Statistical significance was considered at a *p*-value of <0.05.

## Results

### Kinetic Profile of Cytokines

**FIGURE 1:**
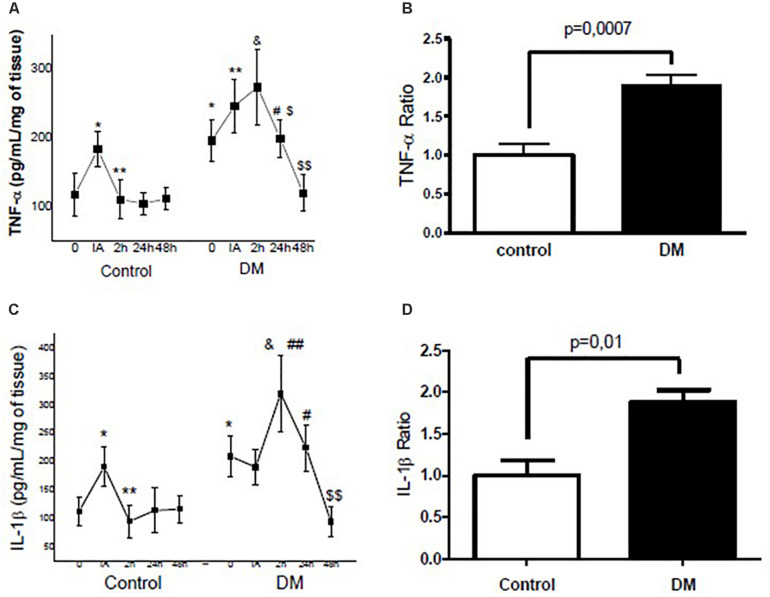
**(A)** Kinetic profile of skeletal muscle tumor necrosis factor (TNF)-α concentrations in the control and diabetic animals evaluated before (0), immediately after (IA), and at 2 h (2), 24 h (24), and 48 h (48) after exercise. **(B)** TNF-α ratio of the integrated area under the curve (IAUC) between the control and diabetic rats. **(C)** Kinetic profile of skeletal muscle interleukin (IL)-1β concentrations in the control and diabetic animals evaluated at 0, IA, and at 2, 24, and 48 h after exercise. **(D)** IL-1β ratio of the IAUC between the control and diabetic rats. The values represent the means ± SE of seven animals per group. ^∗^*P* < 0.05 versus 0 h (control); ^∗∗^ < 0.05 versus IA (control); & < 0.05 versus 2 h (control); # < 0.05 versus 24 h (control); $ < 0.05 versus 2 h (DM); ## < 0.05 versus IA (DM); $$ < 0.05 versus 24 h (DM).

In our assessment of muscle tissue, the levels of TNF-α (0.5-fold; *p* < 0.05) and IL-1β (0.7-fold; *p* < 0.05) were elevated at IA but regressed to basal concentrations at 2 h after exhaustive exercise in the control animals. The response pattern was different in the diabetic group, with the IL-6 (data not shown), TNFα, and IL-1β levels peaking only at 2 h after the exercise protocol and regressing to basal conditions at 48 h after exercise (Figure 1A). Interestingly, before exhaustive exercise, diabetic rats had increased baseline TNF-α (0.8-fold; *p* = 0.05) and IL-1β (1.0-fold; *p* = 0.044) relative to the control group (Figure 1A). Moreover, compared to the control rats, the area under the curve revealed an exacerbated pro-inflammatory response in the diabetic group as demonstrated by increased levels of TNF-α (0.9-fold; *p* = 0.0007) and IL-1β (0.8-fold; *p* = 0.01) (Figure 1B) in the diabetics rats.

**FIGURE 2:**
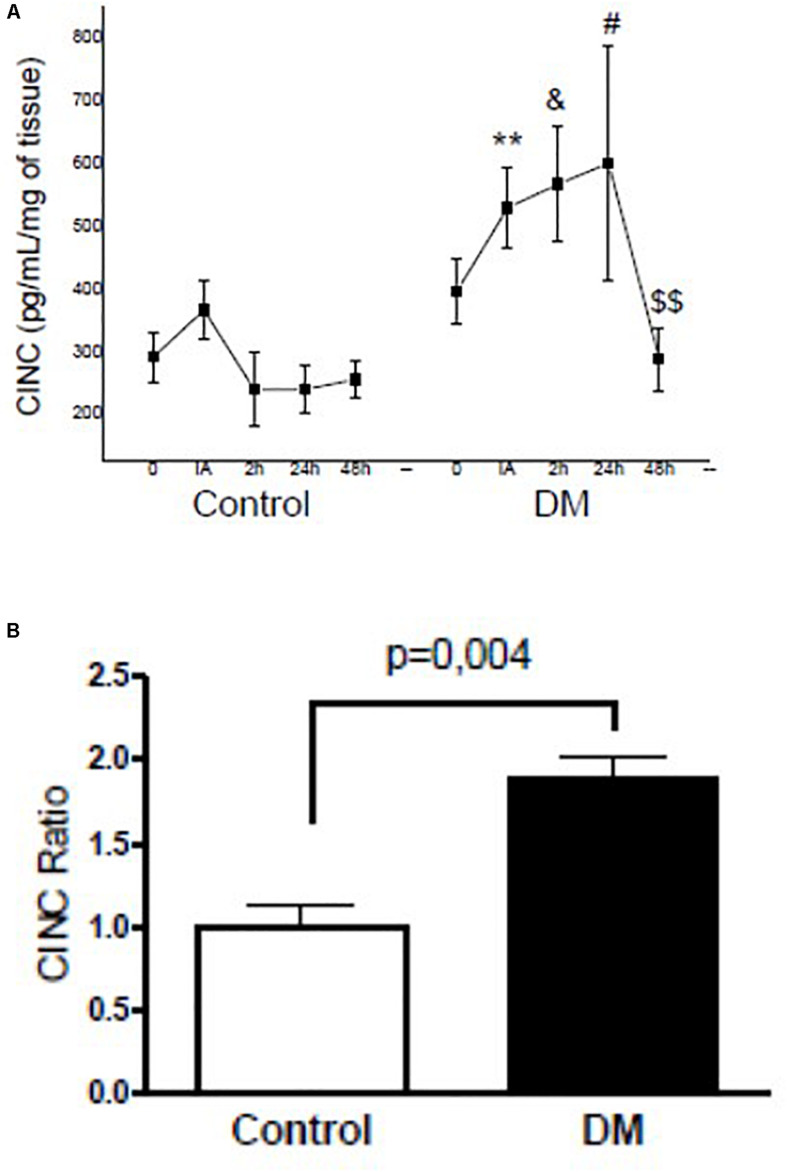
**(A)** Kinetic profiles of the cytokine-induced neutrophil chemo-attractants (CINC) concentration in the skeletal muscle of the control and diabetic animals assessed before (0), immediately after (IA), and at 2 h (2), 24 h (24), and 48 h (48) after exercise. **(B)** CINC ratio of the IAUC between the control and diabetic rats. The values represent the means ± SE of seven animals per group. ** < 0.05 versus IA (control); & < 0.05 versus 2 h (control); # < 0.05 versus 24 h (control); $$ < 0.05 versus 24 h (DM).

**FIGURE 3:**
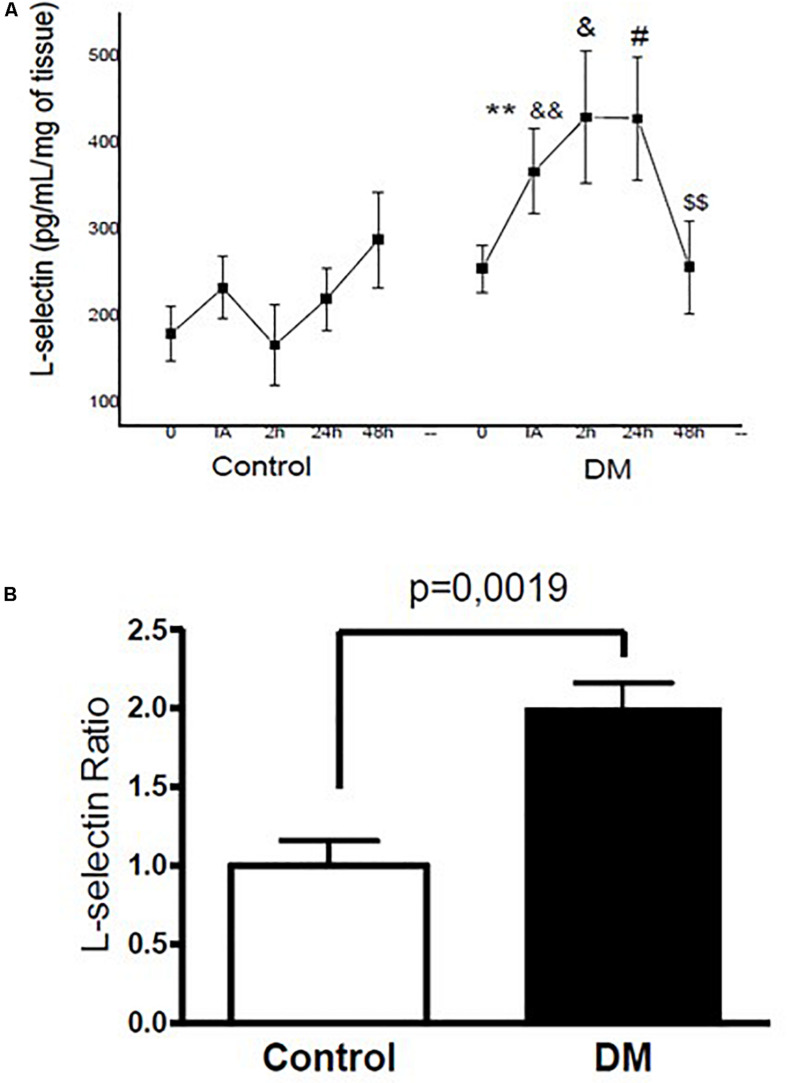
**(A)** Kinetic profiles of the L-selectin concentration in the skeletal muscle of control and diabetic animals assessed before (0), immediately after (IA), and at 2 h (2), 24 h (24), and 48 h (48) after exercise. **(B)** L-selectin ratio of the IAUC between control and diabetic rats. The values represent the means ± SE of seven animals per group. ** < 0.05 versus IA (control); & < 0.05 versus 2 h (control); # < 0.05 versus 24 h (control); && < 0.05 versus 0 h (control); $$ < 0.05 versus 24 h (DM).

Compared to the control group IA, 2 h, and 24 h after exercise, we noted that the muscular levels of CINC (0.7, 0.8, and 1.0-fold; *p* < 0.05, respectively) and L-selectin (1.0, 1.4, and 1.4-fold; *p* < 0.05, respectively) in the diabetic group increased. CINC and L-selectin levels in the diabetic group also returned to baseline values only 48 h after exhaustive exercise (Figures 2A, 3A). Furthermore, the area under the curve also indicated higher concentrations of muscle CINC (0.9-fold; *p* = 0.04) and L-selectin (1.0-fold; *p* = 0.0019) in the diabetic group than in the control group (Figures 2B, 3B).

### Synthesis and Degradation of Muscle Proteins

**FIGURE 4:**
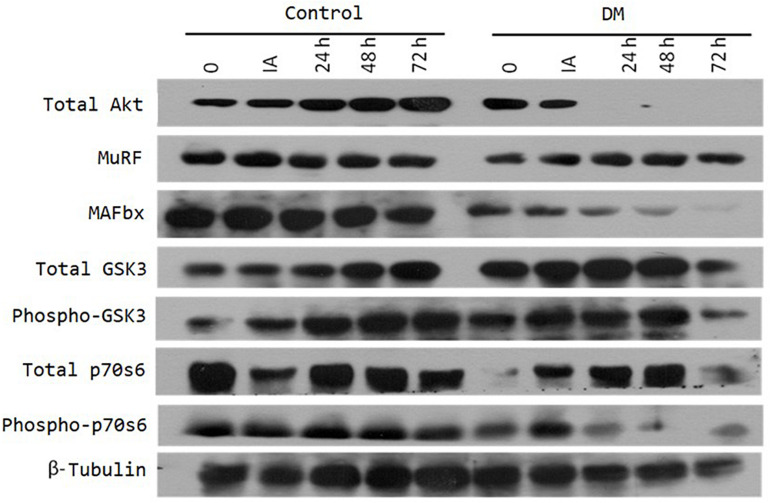
Representative images of Western blots performed with antibodies targeting the following proteins: Akt, MuRF, MAFbx, GSK3, and ap70s6. The soleus muscle of rats from both the control group and the diabetic group were removed before (0), immediately after (IA), and at 2 h (2), 24 h (24), 48 h (48), and 72 h (72) after exercise. Except for Akt, the images represent two experiments that include a pool of four animals per condition. For Akt, the image represents one experiment that included a pool of four animals per condition.

The Akt/mTOR/p70S6k pathway is important in stimulating muscle growth in animals. According to the image obtained from the Western blot experiment (Figure 4), our data showed that p70S6k phosphorylation was decreased in the diabetic group after physical exhaustion. GSK3, a constitutively active kinase that regulates glucose homeostasis and hypertrophy, is inhibited by insulin signaling. In the control animals, we observed increased phosphorylated GSK3 expression over time after physical exhaustion, whereas in the diabetic rates, we observed decreases in total p70S6K and MAFbx expression, p70S6K phosphorylation, and total Akt in the soleus muscle after exhaustive exercise.

## Discussion

Hypertrophy refers to an elevation of myofiber size promoted by intense exercise or anabolic hormones/drugs, and several factors are important contributors to muscle healing after exercise. For example, to optimize exercise interventions, it is important to understand the results of the distinct intensities and types of physical activity on the kinetics of inflammatory mediator release to maximize the beneficial effects of exercise (da Rocha et al., 2019). The ideal form of exercise intervention with the goal of hypertrophy is to potentialize the main event in each stage of the muscle healing progress. To our knowledge, this research was the first to demonstrate the levels of TNF-α, IL-6, IL-1β, L-selectin, VEGF, and CINC-3α/β in the soleus muscle from diabetic rats before exercise, IA exhaustive exercise and at 2 h, 24 h, and 48 h after exercise.

We noted that 2 h after exhaustive exercise, the diabetic animals (but not the control rats) presented elevated levels of muscle TNF-α, IL-1β, IL-6, L-selectin, and CINC. In previous studies, we demonstrated that 24 h after exercise, serum pro-inflammatory cytokines were increased in diabetic animals but not in the control group (Bortolon et al., 2012). We found that inflammation induced by exhaustive exercise were higher and remained longer in animals with diabetes than in non-diabetic control animals. Bortolon et al. (2012) demonstrated that injured muscle fibers release CK and LDH. Under the same conditions in this study, our previous studies indicated that 24 h after exercise, the diabetic rats but not the control animals showed increased serum CK activity (Bortolon et al., 2012). Our data are also in accordance with Galassetti et al. (2006), who showed an altered adaptive pattern to intense physical activity in children with T1DM. They evaluated 12 children with T1DM and 12 control subjects aged 11–15 years and applied a 30-min exercise stimulus at 80% of the VO_2max_. Compared to the control children, the IL-6 levels were higher in T1DM children, suggesting that T1DM children display significant alterations in the adaptive response to intense exercise (Galassetti et al., 2006). Rosa et al. (2008) also studied children with T1DM and healthy control subjects who engaged in 30 min of intense and intermittent cycling exercise and found that compared to the control subjects, T1DM children exhibited an earlier and greater exercise-induced IL-6 peak, and they also noted an exaggerated increase in the kinetic profiles of additional inflammatory mediators in children with T1DM (Rosa et al., 2008).

Increased baseline levels of TNF-α, IL-8, and IL-6 have been observed in diabetics (Erbagci et al., 2001; Mohamed-Ali et al., 2001). Interestingly, *in vitro* studies also noted a similar pattern when investigating cytokine production in suspensions of cell cultures, with excessive release of IL-8, IL-1β, and TNF-α by neutrophils and mononuclear cells (Hatanaka et al., 2006). However, although the baseline values observed in this study are similar to that of the above studies, this study was performed to investigate the kinetics of these inflammatory mediators and demonstrate the exact period of the return to baseline. Our data indicate that 24 h after exhaustive exercise was sufficient for all the inflammatory markers (except for L-selectin and CINC) to return to baseline in the diabetic animals. In addition, we found that at 48 h, the diabetic rats exhibited a significant reduction in inflammatory markers and reached values similar to those found in non-diabetic animals subjected to the same exercise regimen.

Protein signaling for synthesis and degradation (phosphorylated S6 and MAFbx, respectively) were suppressed in the skeletal muscle of diabetic rats, which indicates a possible suppression of protein turn-over in the skeletal muscle of diabetic rats. Since intracellular toll-like receptors are relevant influencers of muscle function and important regulators of muscle fiber size and tissue inflammation (De Paepe, 2020), the excessive inflammation found in our data might also be related to the protein signaling changes noted post-exercise. The excessive inflammation noted in wound tissue in individuals with diabetes can advance the delays in wound healing often observed in this population. The treatment of diabetic muscle atrophy lacks a pharmacological target, and diabetes can lead to a chronic low-level inflammation status, thereby inducing atrophy and negatively influencing muscle healing (Gurtner et al., 2008; Pence and Woods, 2014). Because the inflammatory markers from global blood circulation can also occur in skeletal muscle, future studies should also investigate the level of muscle damage by muscle-specific creatine kinase (CK-MM) analysis after exhaustive exercise.

Decreasing muscle mass is a predictor of morbidity and mortality in individuals with diabetes, and it is associated with poor diabetic control, physical inactivity, and chronic inflammation (Furrer and Handschin, 2019). Moreover, exercise is associated with a marked increase in the phosphorylation of several proteins involved in atrophy and hypertrophy signal transduction (Marcotte et al., 2015). However, although many studies have focused on exercise and diabetes and their effects on one another, very few studies have addressed the effects of exercise on the kinetic muscle cytokines related to the healing process and signaling relating to atrophy and hypertrophy.

MAFbx and MuRF-1 are induced by inflammatory signaling pathways (MAPK, FOXO, and NF-κB). Our data showed that exhaustive exercise decreased MAFbx expression in the diabetic group compared to the control group in a time-dependent manner. The decreased activation ratios of MAFbx are in accordance with decreases in inflammatory factors, such as TNF-α, IL-1β and IL-6, which have been shown to activate pathways that regulate MuRF-1 and MAFbx expression (Sishi and Engelbrecht, 2011; Lin et al., 2016).

For diabetic patients without major complications, the American Diabetes Association and the American College of Sports Medicine suggest regular resistance and aerobic exercise (American Diabetes Association, 2009; Dugan, 2016; Association, 2020). However, physical training followed by inflammation may be harmful to the health of people with diabetes; moreover, the guidelines applied for prescribing physical activity for this public do not provide information on the exercise intensity for diabetic patients based on their inflammatory profile. Our data indicated that after an exhaustive exercise session, despite both groups showing recovery of the inflammatory response, diabetic animals recovered more slowly than control rats and showed a more effective return of inflammatory cytokines to baseline levels. The main findings of our study are that marked differences occur in the kinetic inflammatory profile between diabetic and non-diabetic animals in relation to the repair of skeletal muscle and their comprehensive adaptive responses to an exhaustive exercise protocol and these different inflammatory behaviors can modulate atrophy and hypertrophy signaling in muscle.

## Data Availability Statement

The raw data supporting the conclusions of this article will be made available by the authors, without undue reservation.

## Ethics Statement

The animal study was reviewed and approved by the Ethics Committee on Animal Experimentation at Universidade Cruzeiro do Sul.

## Author Contributions

EH and JB were involved in the concept and design. JB, MS, GM, and AD were involved in the acquisition of data. JB and EH analyzed the data. LB, AD, EW, and EH drafted the manuscript and performed a critical revision of the study. All authors reviewed and approved the final manuscript.

## Conflict of Interest

The authors declare that the research was conducted in the absence of any commercial or financial relationships that could be construed as a potential conflict of interest.
